# Supplementary material for: Mapping the evolution of fertility support policies in China: A content and instrumental analysis
Source: PLoS One. 2025 Oct 9;20(10):e0332137. doi: 10.1371/journal.pone.0332137 (PMC12510515; doi:10.1371/journal.pone.0332137)
Supplement: S1 Appendix — (ZIP) [file pone.0332137.s001.zip › S1 Appendix. 226 original policy documents/117-人事部关于机关、事业单位女职工产假期间工资计发问题的通知(FBM-CLI-4-37017).doc]

人事部关于机关、事业单位女职工产假期间工资计发问题的通知

**发布部门：** [人事部(已撤销)](https://www.pkulaw.com/law/chl?Aggs.IssueDepartment=60211) [机构沿革](javascript:void(0))

**发文字号：**人薪发〔1994〕7号

**发布日期：**1994.02.24

**实施日期：**1993.10.01

**时效性：** [现行有效](https://www.pkulaw.com/law/chl?Aggs.TimelinessDic=01)

**效力级别：** [部门规范性文件](https://www.pkulaw.com/law/chl?Aggs.EffectivenessDic=XE0302)

**法规类别：** [工资福利与劳动保险](https://www.pkulaw.com/law/chl?Aggs.Category=08707)

　　　　人事部关于机关、事业单位女职工产假期间工资计发问题的通知
　　　　　　　　　　 　（人薪发〔１９９４〕７号）

各省、自治区、直辖市及计划单列市人事（劳动人事）厅（局），国务院各部委、各直属机构人事（干部）司（局），新疆生产建设兵团：
　　关于机关和事业单位工资制度改革后，女职工产假期间工资如何计发问题，经研究，通知如下：
　　**一、**机关、事业单位工资制度改革后，女职工在国家规定的产假期间，其工资按下列各项之和计发；
　　１、机关实行职级工资制的人员，为本人职务工资、级别工资、基础工资与工龄工资；
　　２、机关技术工人，为本人岗位工资、技术等级（职务）工资与按国家规定比例计算的资金；
　　３、机关普通工人，为本人岗位工资与按国家规定比例计算机的奖金；
　　４、事业单位职工，为本人职务（技术等级）工资与按国家规定比例计算机的津贴（其中，体育运动员，为本人体育基础津贴与成绩津贴）。

　　**二、**本通知自１９９３年１０月１日起执行。
　　　　　　　　　　　　　　　　　　　　　　　　　　１９９４年２月２４日
c60025--010601ljk

© 北大法宝：（[www.pkulaw.com](https://www.pkulaw.com/)) 专业提供法律信息、法学知识和法律软件领域各类解决方案。北大法宝为您提供丰富的参考资料，正式引用法规条文时请与标准文本核对。
欢迎查看所有[产品和服务](https://www.pkulaw.com/helps/2.html)。法宝快讯：[如何快速找到您需要的检索结果？](https://www.pkulaw.com/helps/70.html)[法宝](https://www.pkulaw.com/helps/69.html)V6有何新特色？

扫描二维码阅读原文

**原文链接：**<https://www.pkulaw.com/chl/ceaf4e6545ee0ed4bdfb.html>
